# 608. Continuous Infusion Vancomycin Is Not Associated with Improved Safety in an Outpatient Parenteral Antimicrobial Therapy Program

**DOI:** 10.1093/ofid/ofab466.806

**Published:** 2021-12-04

**Authors:** Rosemarie D Tagare, Joshua A McDonald, Brandon Tritle, Karen Fong, Michael G Newman, Laura Certain, Russell J Benefield

**Affiliations:** 1 University of Utah Health, Salt Lake City, Utah; 2 Intermountain Healthcare, Salt Lake City, Utah; 3 University of Utah, Salt Lake City, Utah

## Abstract

**Background:**

Continuous infusion (CI) vancomycin has been reported to be associated with improved safety outcomes compared to intermittent infusion (II) in the outpatient parenteral antimicrobial therapy (OPAT) setting. Based on this our institution implemented a quality improvement intervention to discharge more patients on CI vancomycin aiming to improve vancomycin safety in our OPAT program.

**Methods:**

This single-center, pre-/post-intervention, quasi-experimental study evaluated adult patients who received vancomycin for a minimum 7-day intended duration of therapy after discharge, were discharged to home health or a skilled nursing facility, and had a follow-up visit with an infectious diseases provider. Outcomes included discontinuation due to acute kidney injury (AKI) or due to any adverse drug event (ADE), time to AKI or ADE, and unplanned 30-day readmissions and were compared between the pre-intervention (11/25/2018 to 7/5/2020) and post-intervention (7/6/2020 to 3/31/2021) periods. Adverse events were defined as premature discontinuation of vancomycin with documentation of a suspected adverse event.

**Results:**

Of the 445 patients included, 102 patients received CI vancomycin. Demographic characteristics were generally similar between time periods, although more patients discharged to home health were included during the post-intervention period. CI vancomycin use was higher after the intervention (42% vs 11%, P < 0.0001). Discontinuation due to AKI (7% vs 8%, P = 0.68) or any ADE (16% vs 18%, P = 0.65) occurred just as frequently post-implementation. Unplanned 30-day readmission was higher post-intervention (21% vs 12%, P = 0.02). When comparing patients receiving CI and II vancomycin, discontinuation rates due to AKI (10% with CI vs 7% with II, P = 0.35) and any ADE (17% with CI vs 17% with II, P = 0.85) were similar. Time to AKI (median 21 days with CI vs 16 days with II, P = 0.26) and any ADE (median 22 days vs 22 days, P = 0.55) were also similar. There was a trend toward a significantly higher unplanned 30-day readmission rate with use of CI compared to II (22% vs 14%, P = 0.07).

Control Charts

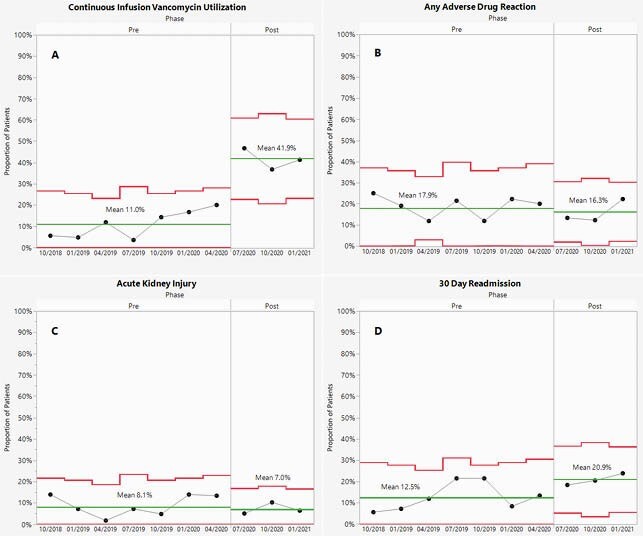

These control charts show the variation over time of the proportion of patients A. utilizing CI vancomycin, B. experiencing any adverse drug reaction, C. experiencing acute kidney injury, and D. being readmitted within 30 days. Upper and lower control limits are depicted by red lines, and the mean is depicted by a green line.

**Conclusion:**

We found no safety advantages when using CI instead of II vancomycin in the outpatient setting. The potentially higher readmission rate observed with CI vancomycin will be investigated further.

**Disclosures:**

**Russell J. Benefield, PharmD**, **Paratek Pharmaceuticals** (Grant/Research Support)

